# Supplementary material for: miR‐515‐5p controls cancer cell migration through MARK4 regulation
Source: EMBO Rep. 2016 Feb 10;17(4):570–84. doi: 10.15252/embr.201540970 (PMC4818771; doi:10.15252/embr.201540970)

APPENDIX

TABLE OF CONTENT

**Tables and Figure legends**	**PAGE 2**
**Appendix Table S1**	**PAGE 5**
**Appendix Table S2**	**PAGE 5**
**Appendix Table S3**	**PAGE 5**
**Appendix Figure S1**	**PAGE 7**
**Appendix Figure S2**	**PAGE 8**
**Appendix Figure S3**	**PAGE 9**
**Appendix Figure S4**	**PAGE 10**
**Appendix Figure S5**	**PAGE 11**
**Appendix Figure S6**	**PAGE 12**
**Appendix Figure S7**	**PAGE 13**
**Appendix Figure S8**	**PAGE 14**
**Appendix Figure S9**	**PAGE 15**
**Appendix Figure S10**	**PAGE 16**
**Appendix Figure S11**	**PAGE 17**
**Appendix Figure S12**	**PAGE 18**
**Appendix Figure S13**	**PAGE 19**

APPENDIX TABLES AND FIGURE LEGENDS

Appendix Table S1. Sequence of MARK4 siRNAs (Dharmacon).

Appendix Table S2. Sequence of qRT–PCR primers**.**

Appendix Table S3. Clinico-pathological information of breast cancer patients.

LEGENDS

Appendix figure S1: (A-B) The indicated cell lines were either transfected with a non-targeting control (NC) or a miR515-5p mimetic and intracellular levels of miR515-5p determined 48h later by Taqman-based qPCR (A) or caspase 3/7 activity determined using a CaspaseGlo assay (B). (A) Data were normalized to u6 snRNA values and the mean of quadruplicates ± SEM. (B) Data are average of triplicate ± SEM of triplicate. (A-B) P values were calculated by t-test (***, P<0.001; n.s., non-statistically significant). Data shown are representative of experiments performed in triplicate.

Appendix figure S2: Overexpression of miR-515-5p leads to a loss of cell polarity. Breast (MDA 231 and MCF7) and lung (A549 and H1299) cancer cells were transfected with miR-515-5p mimics or control non-targeting miRVana prior to actin cytoskeleton staining and fluorescent microscopy image acquisition. The circularity factor was determined for individual cells and represented as a dot plot. Ten fields of view were analysed per condition. Horizontal bars: median value. Statistical analysis: ANOVA (***, P<0.001). Data shown are representative of experiments performed in triplicate.

Appendix figure S3: The effect of miR-515-5p sponges in NRAS, FZD4, CDC42BPA, PIK3C2B and MARK4 mRNA levels in MCF7 (A) and MDA-MB-231 (B). The miR-515-5p sponges were constructed by annealing, purifying and cloning oligonucleotides containing six tandem bulged miRNA binding motifs, into the HindIII and BamHI sites of the pEGFP-C1 plasmid (Contech, Saint-Germain-en-Laye, France). After 48 h of miR-515-5p sponges transfection, NRAS, FZD4, CDC42BPA, PIK3C2B and MARK4 mRNA levels were quantified by Syber Green qRT-PCR. Data were normalized to U6 snRNA values and are presented as the mean ± SEM of quadruplicates. P values were calculated by t-test comparing individual miR-515-5p sponges values with NC sponges values (**,P<0.01; ***,P<0.001). Data shown are representative of experiments performed in triplicate.

Appendix figure S4: miR-515-5p expression in MCF7 and MDA-MB-231. miR-515 levels were quantified by Taqman qRT-PCR. Data were normalized to U6 snRNA values and are presented as the mean ± SEM of quadruplicates. Data shown are representative of experiments performed in triplicate.

Appendix figure S5: Luciferase reporter assay after co-transfection of MDA231 cells with hsa-miR-515-5p mimic and switchgear-3'UTR-MARK4 (Active Motif) reporter vector wild-type or mutated for the predicted sites A and B-miR-515-5p interaction sites. Bar graphs are the average ± SEM of triplicate. Data shown are representative of experiments performed in triplicate. The positions of the point mutations in the MARK4 UTR are shown above the bar graph and the corresponding sequences displayed underneath it. Data shown are representative of experiments performed in triplicate.

Appendix figure S6: (A) Efficient silencing of MARK4. The indicated cell lines were treated either with a MARK4 or a not targeting (NT) siRNA pool. Levels of MARK4 mRNA were determined 48h later by qPCR. Bar graphs are average of n=3 ± SEM normalised to the corresponding NT condition. (B-C) MDA157 cells were transfected with a MARK4, a not targeting (NT) siRNA pool, miR515-5p or a

corresponding non-targeting miR (NC). (B) Random cell migration assays were performed over 18 hour by live cell imaging and the distance covered by migrating cells determined by cell tracking. Data shown are average normalised measurements from n=30 cells per condition. (C) Cells were fixed and stained for nuclear DNA (DAPI, blue) and tubulin (green). Scale bar: 200µm. 27 fields per condition were acquired (x20) and the average tubulin area per cell calculated using Fiji and the fold changes ± SEM plotted. (B-C) Statistical analysis: ANOVA (**,P<0.01; ***,P<0.001). Data shown are representative of experiments performed at least in triplicate.

Appendix figure S7: (A) The indicated cell lines were transfected with either an empty vector (EV) or a MARK4 expressing plasmid DNA. MARK4 mRNA levels were determined 48h later. (B-C) The indicated cell lines were co-transfected with either miR515-5p or a corresponding non-targeting miR (NC) in the presence or absence of a MARK4 expressing (pMARK4) or an empty vector (EV) plasmid DNA and random cell migration (B) or transwell assays conducted. (A) Bar graphs show average of technical triplicate ± SEM. (B) Cell tracks of n=30 cells. (C) Crystal violet stained cells pictured on the bottom-side of the transwell filter. Results shown are representative of experiments performed in triplicate.

Appendix figure S8: MARK4 silencing leads to cell cycle arrest. A549 cells were transfected with MARK4 siRNA (Pool for (A) and (B) or individual targeting sequences 1 to 4 for (B)) or non-targeting sequences (NT). 48 hours later, the cells were fixed and stained with Click-iT EdU prior to flow cytometric analysis (A) or the cell lysates subjected to SDS-PAGE/Western blotting for the indicated proteins. (A) The data were analysed in FlowJo for determining the proportion of cells in each phase of the cell cycle. Statistical analysis: ANOVA (*, P<0.05). Data shown are representative of at least three independent experiments (A and B) performed in triplicate (A).

Appendix figure S9: Change in tubulin polymerization speed. A549 expressing an EB3-GFP construct cells were transfected with MARK4 siRNA or non-targeting sequences (NT) and fast image acquisition performed using a Zeiss LSM-780 inverted confocal microscope using the Zen Black software (Zeiss). (A) Representative images of microtubule growth. (B) Quantification of the speed of microtubule growth. Comet speed was analysed using FIJI ImageJ (NIH, USA) and the plugin wrMTrck. Bar graphs are the average of triplicate experiments with n=12 cells per condition analysed in each experiment. Error bars indicate SEM. Asterisks represent statistically significant values from a minimum of three biological repeat experiments with three technical repeats (**, P<0.01; Students T Test).

Appendix figure S10: (A-B) Distant relapse-free survival of ER-negative metastatic breast (A) and overall survival of metastatic lung (B) cancer patients according to the levels of miR-515-5p. GEO2R analysis of miR-515-5p expression and survival using the dataset GSE22216 (ERα-negative lymph node-positive breast cancer patients, n=18) [26] and GSE16025 (lymph node-positive lung cancer patients, n=20) [21]. Survival values were divided in two cohorts by a Kaplan-Meier plot using GraphPad Prism: high miR-515-5p expression (> 8.20) and low miR-515-5p expression (< 8.20).

Appendix figure S11: Pearson correlation (r) was computed between the expression of miR-515-5p and MARK4 in various breast cancer subtypes from the TCGA breast cancer dataset.

Appendix figure S12: miR-515-5p expression of primary tumor and metastatic cells in breast cancer patients. RNA was isolated from 18 breast cancer tumor and metastatic tissues. miR-515-5p levels

were quantified by TaqMan qRT-PCR and analyzed either by comparing the paired values for each patient.

Appendix figure S13: NRAS and PI3KC2B expression of primary tumor and metastatic MDA-MB-231 cells in vivo. MDA-MB-231 cells were inoculated into the mammary fat pads of nude mice and allowed to form metastatic deposits. The levels of NRAS (A) and PI3KC2B (B) of both primary tumor and metastatic cells were then quantified by Syber Green qRT-PCR. Data were normalized to U6 snRNA values and are presented as the mean of triplicates ± SEM. P values were calculated by t-test comparing miR-515-5p values in metastasis with values in primary tumour (**,P<0.01). Results shown are representative of experiments performed in triplicate.

Appendix Table S1. Sequence of MARK4 siRNAs (Dharmacon).

Supplier	Target Gene	Dharmacon siGENOME siRNA ID	Sequence
Dharmacon	*MARK4*	D-005345-06	GCUGUACUCUCGAGCAAAU
		D-005345-05	GGAUCAACAUCGGCUAUGA
		D-005345-02	GGAAGUACCGGGUCCCUUU
		D-005345-01	GAUCGAAGCUGGACACGUU

Appendix Table S2. Sequence of qRT–PCR primers**.**

Target Gene	Forward primer sequence	Reverse primer sequence
NRAS	TGTGATTTGCCAACAAGGAC	CAACACCCTGTCTGGTCTT
PI3KC2B	TACCTCGTCCATCTCCAAGA	GGAAGTCTCCATCAGCCAG
FZD4	CAACGTGACCAAGATGCC	AGGAAGAACTGCAGCTGG
CDC42BPA	GTGATTGGTCGAGGAGCT	ACATGCTGTCTCAGCTCTTT
MARK4	GTCAACAGACTGTGAGAGCATCC	GCTCTGTGTATGGCTTCAACTCC
GAPDH	AGCCACATCGCTCAGACAC	GCCCAATACGACCAAATCC
HPRT	TGACACTGGCAAAACAATGCA	GGTCCTTTTCACCAGCAAGCT

Appendix Table S3. Clinico-pathological information of breast cancer patients.

Patients	Sex	Age	Type of Ca	Grade	ER	PgR	Her2	EGFR
1	F	50	IDC	3	-	-	3+	-
2	F	94	IDC	2	-	-	-	+
3	F	33	IDC	2	-	-	-	-
4	F	57	IDC	3	-	1+	3+	-
5	F	68	IDC	3	-	-	-	+
6	F	59	IDC	2	-	1+	-	-
7	F	52	IDC	3	-	-	-	+
8	F	45	IDC	3	-	-	3+	+
9	F	57	IDC	3	-	-	3+	-
10	F	79	IDC	2	-	1+	-	+
11	F	70	IDC	2	-	1+	-	-
12	F	44	IDC	3	-	1+	3+	-
13	F	39	IDC	3	2+	2+	-	+
14	F	44	IDC	3	1+	1+	3+	+
15	F	40	IDC	3	1+	-	-	-
16	F	64	IDC	3	1+	-	-	+
17	F	47	ILC	2	3+	3+	-	-
18	M	62	IDC	2	3+	2+	2+	-

APPENDIX FIG S1

A

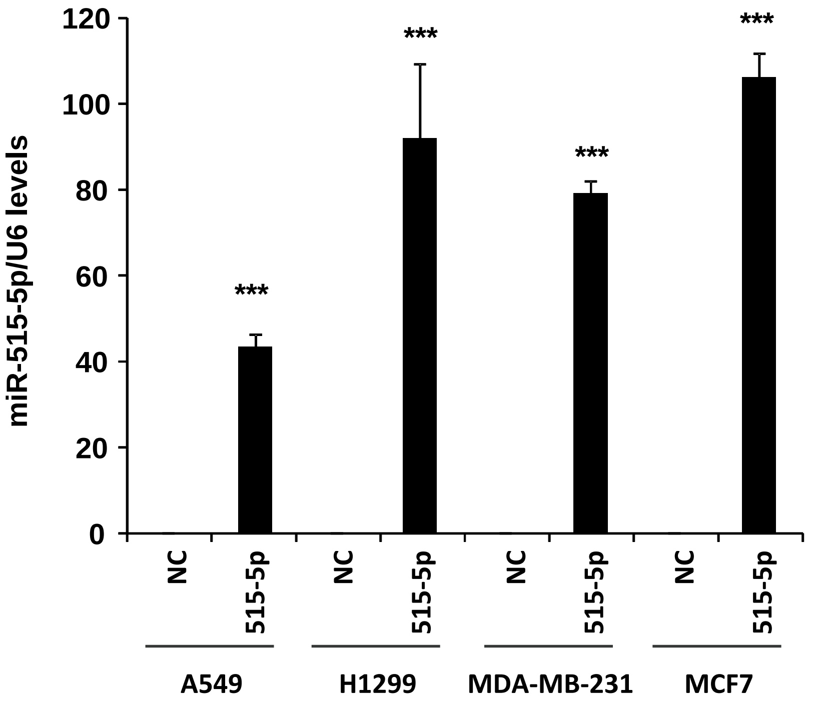

B

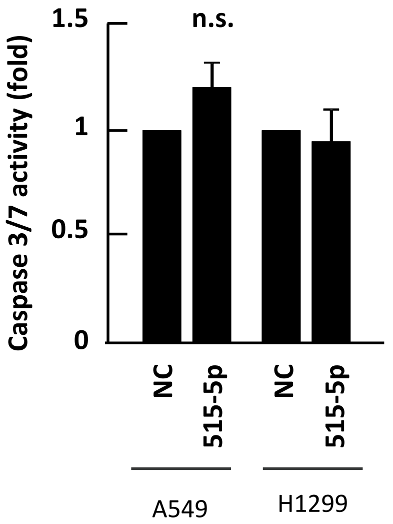

APPENDIX FIG S2

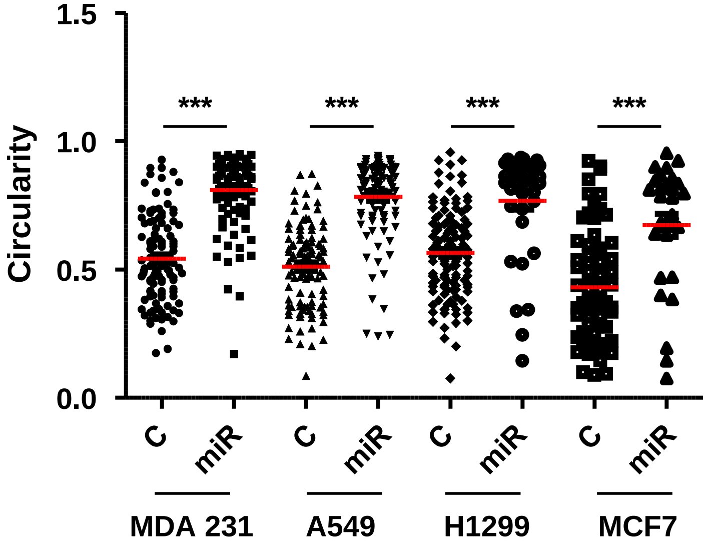

APPENDIX FIG S3

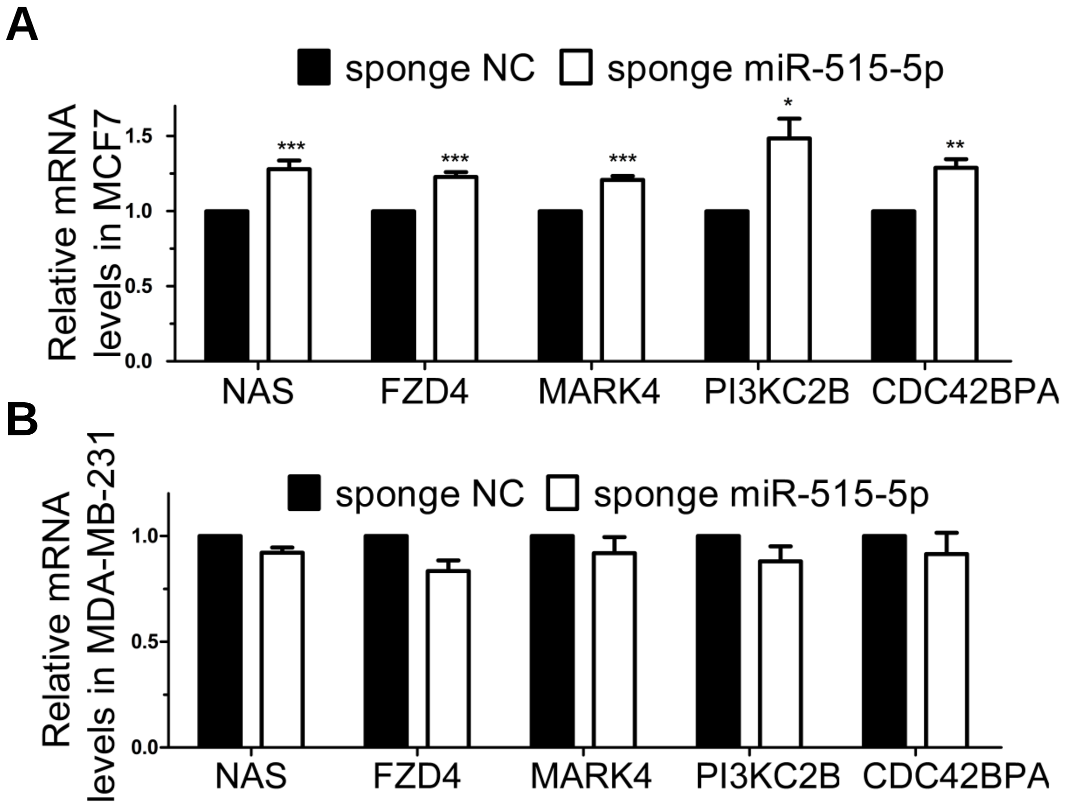

APPENDIX FIG S4

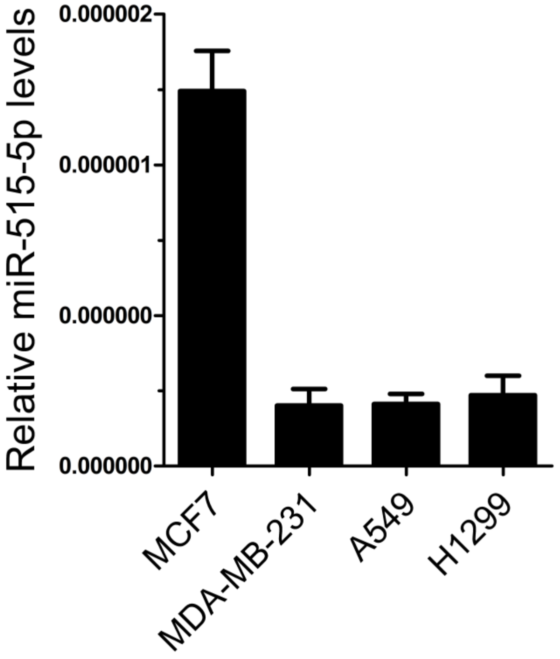

APPENDIX FIG S5

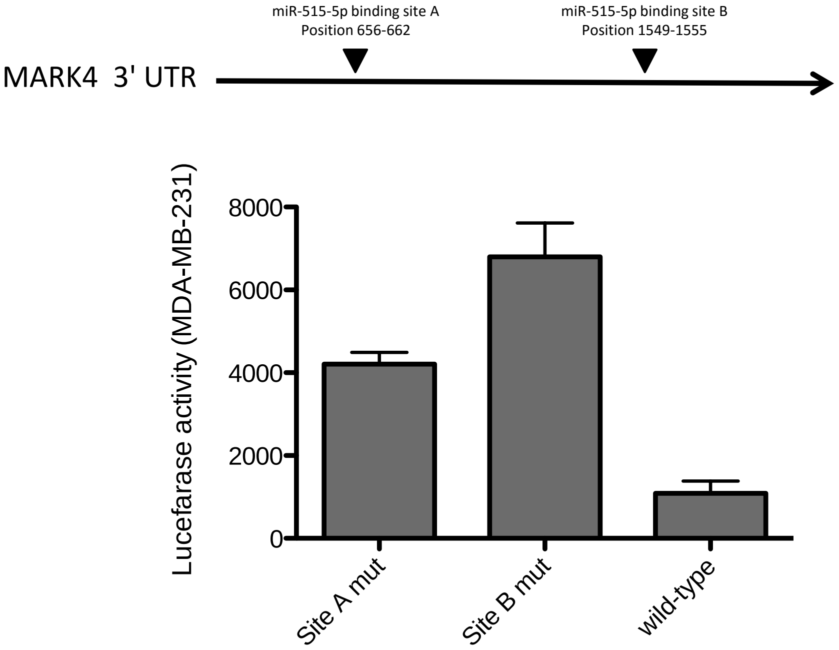


```
5'    ...GCUGAGCCUAAAGACUGGAGAAU...               MARK4 3' UTR A wild-type
                          ||||||
3'     GUCUUUCACGAAAGAAAACCUCUU miR-515-5p
                          ||    |
5'    ...GCUGAGCCUAAAGACUGCUCUAU...               MARK4 3' UTR A mut
```

```
5'    ...UGUGUUGGGCAUGGAUGGAGAAU...               MARK4 3' UTR B wild-type
                          ||||||
3'     GUCUUUCACGAAAGAAAACCUCUU miR-515-5p
                          ||    |
5'    ...UGUGUUGGGCAUGGAUGCUCUAU...               MARK4 3' UTR B mut
```

APPENDIX FIG S6

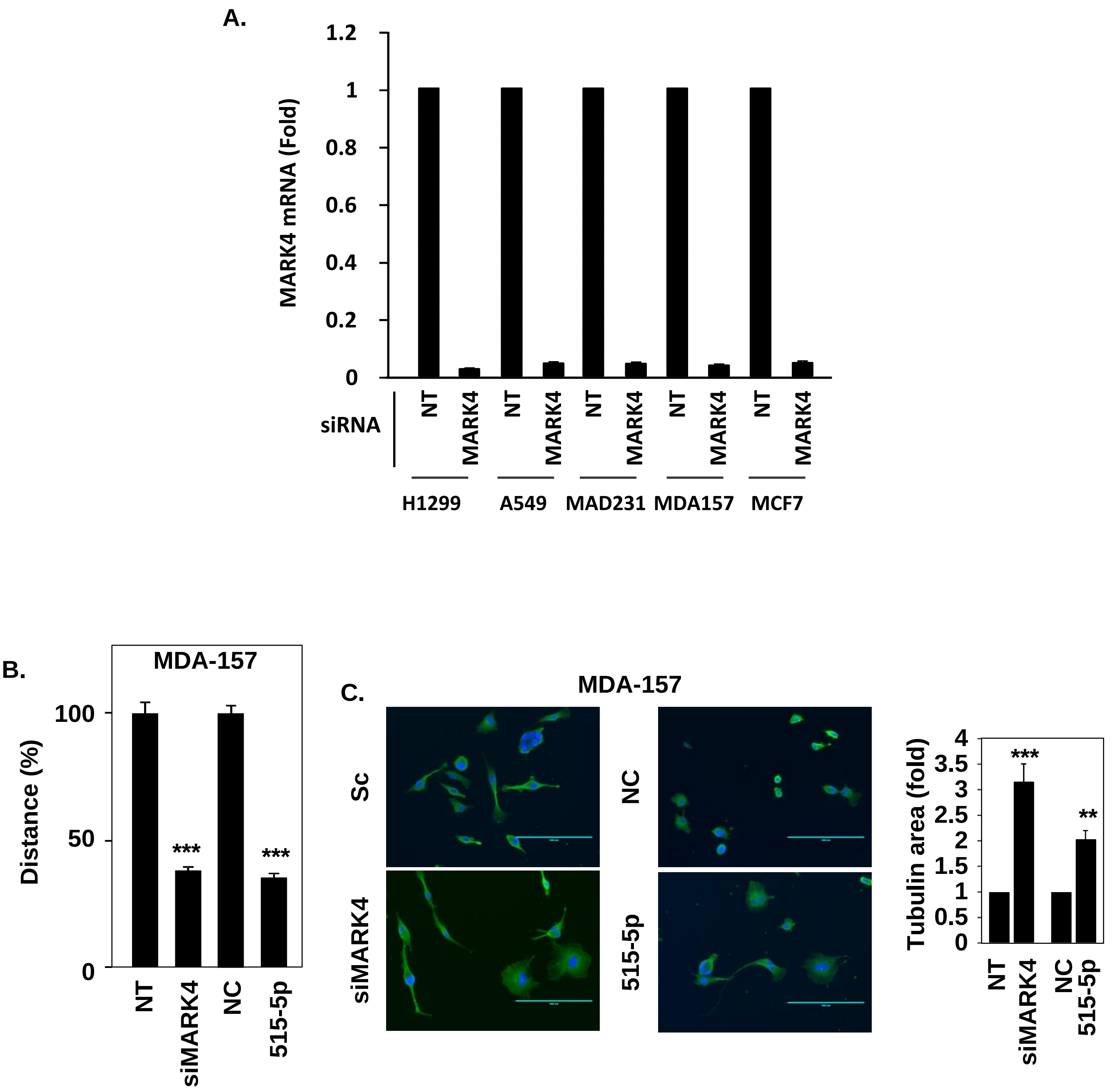

APPENDIX FIG S7

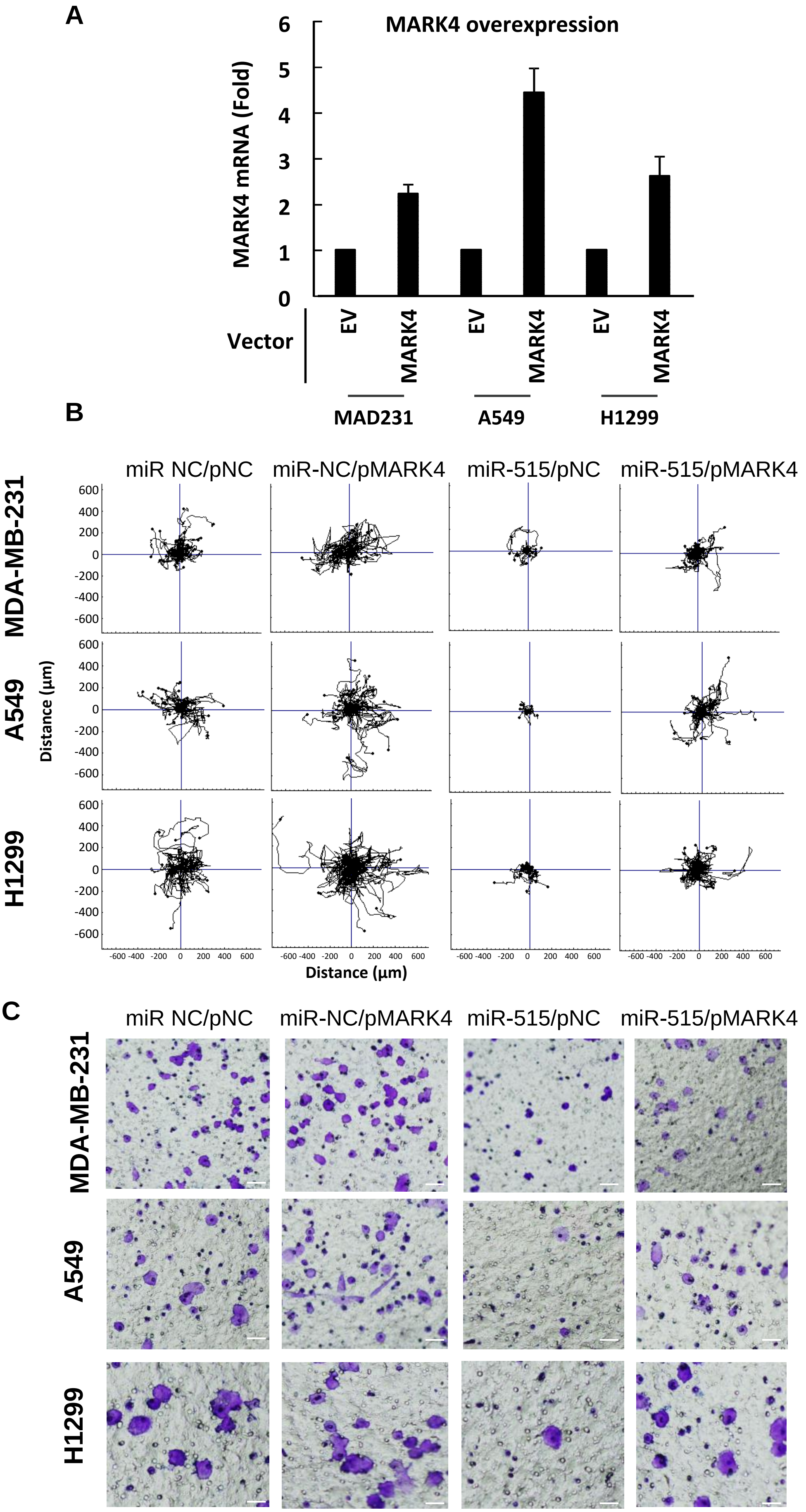

APPENDIX FIG S8

A.

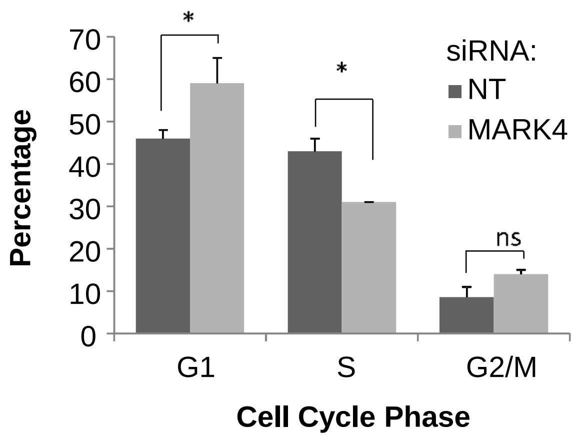

B.

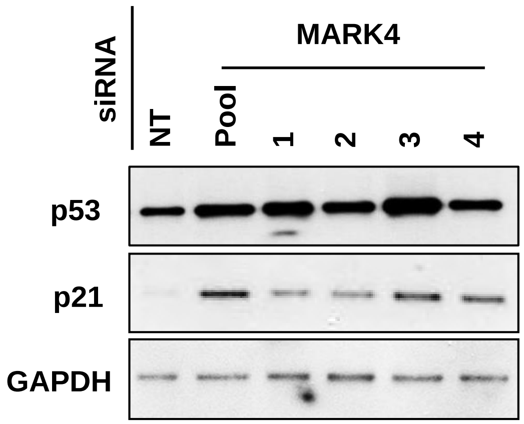

APPENDIX FIG S9

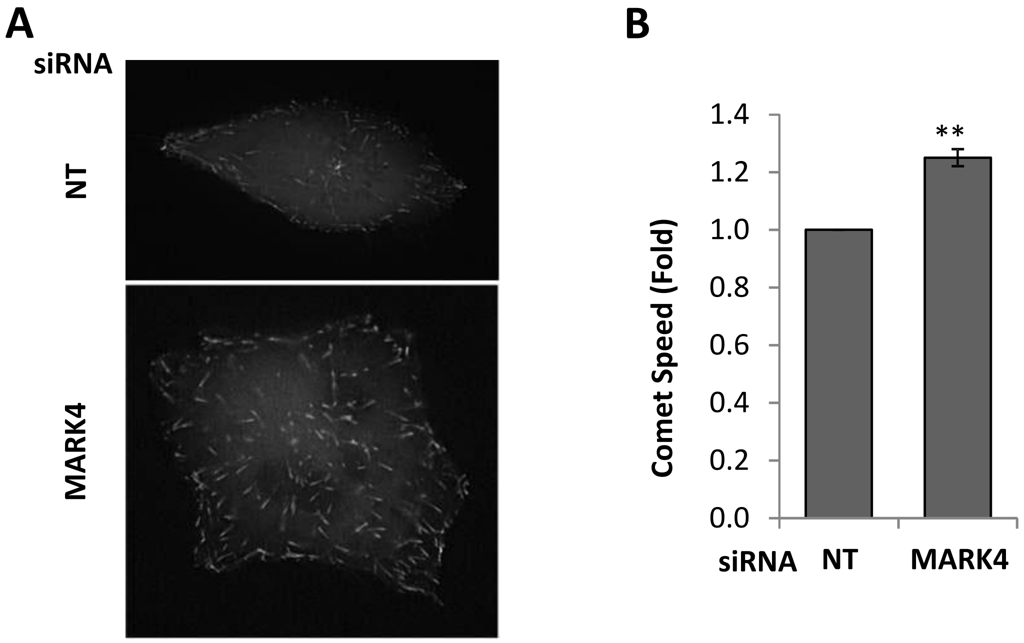

APPENDIX FIG S10

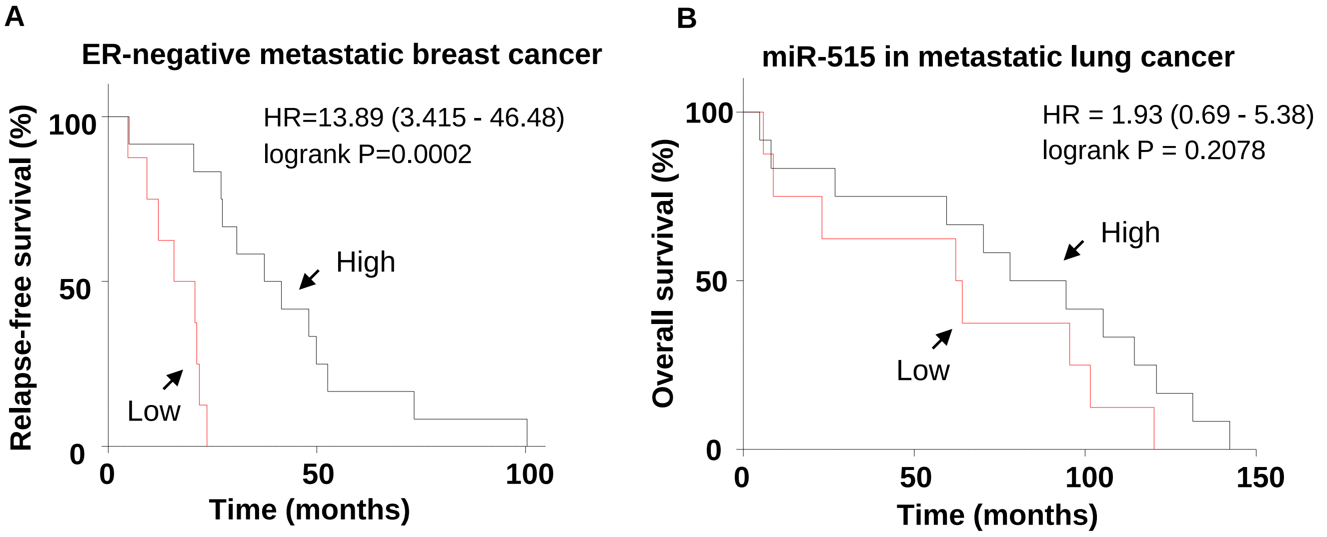

APPENDIX FIG S11

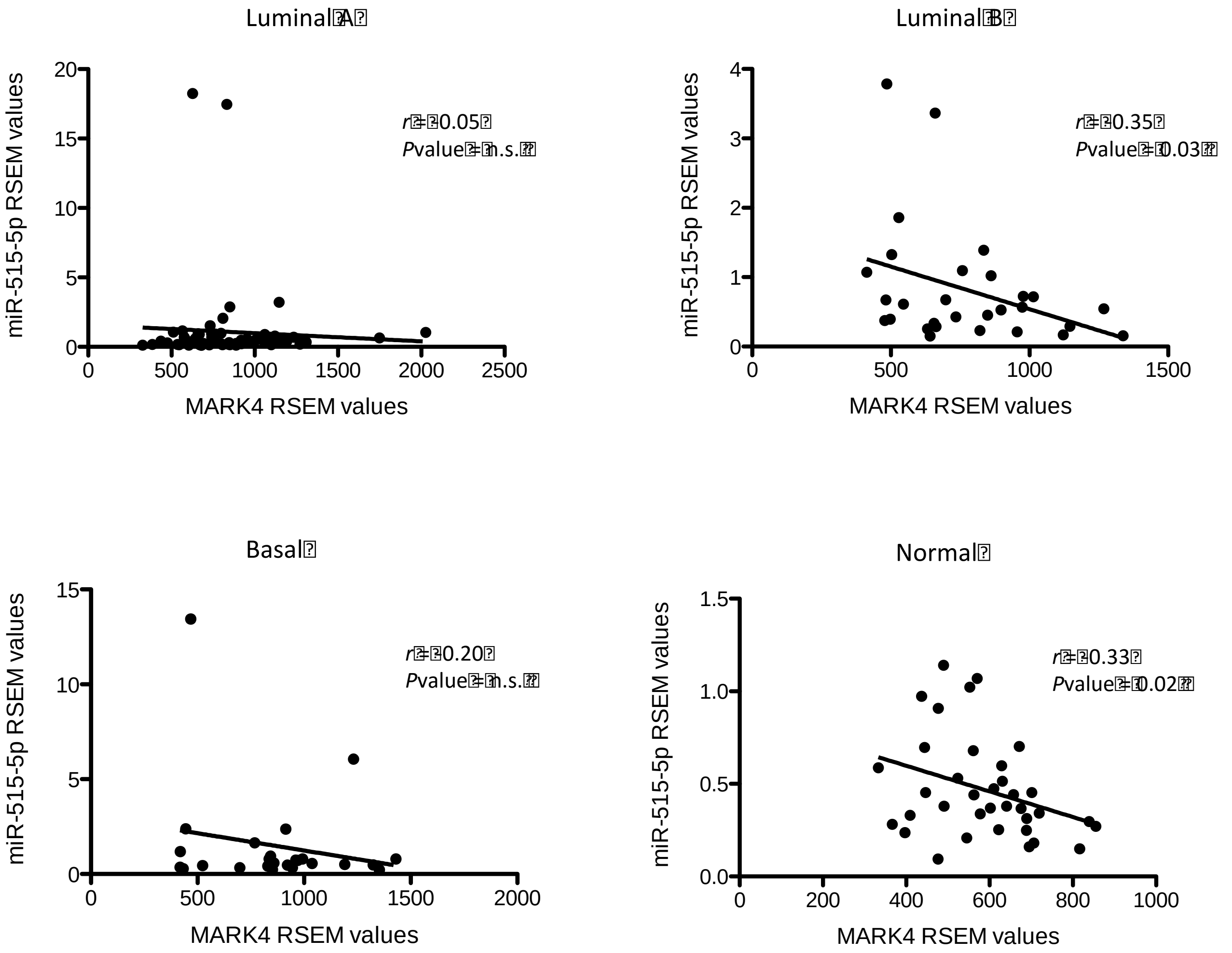

APPENDIX FIG S12

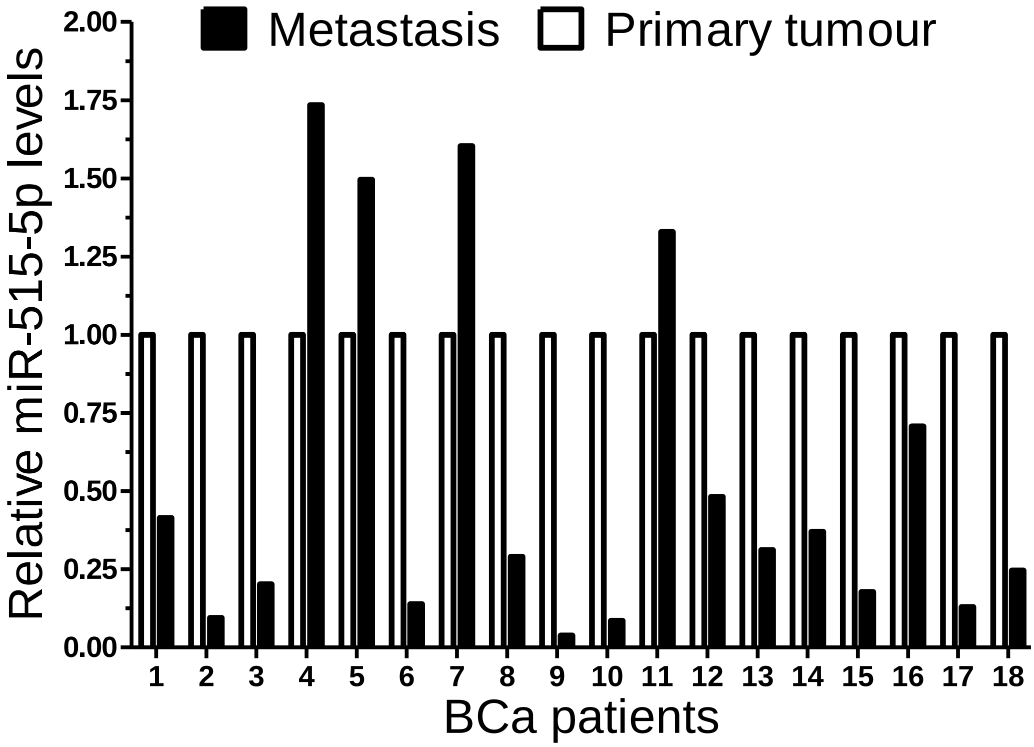

APPENDIX FIG S13

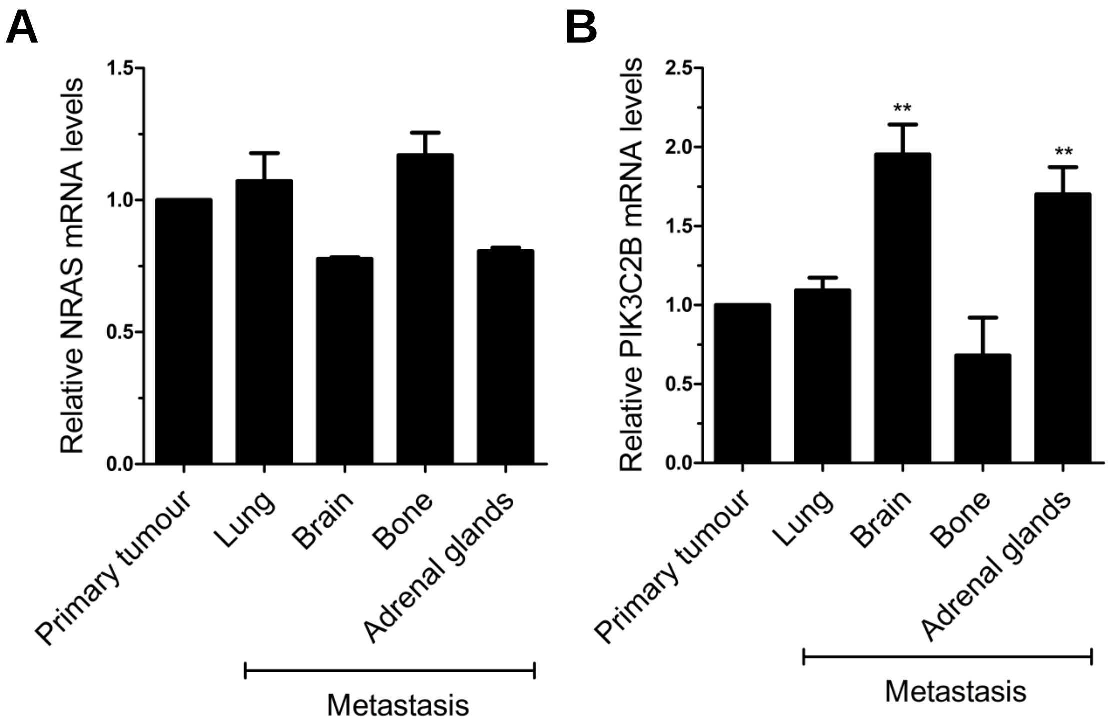

Supplement: Supplementary file 1 — Appendix [file EMBR-17-570-s001.pdf]
